# Satisfied quantitative value can be acquired by short-time bone SPECT/CT using a whole-body cadmium–zinc–telluride gamma camera

**DOI:** 10.1038/s41598-021-03853-0

**Published:** 2021-12-21

**Authors:** Tomohiko Yamane, Masafumi Takahashi, Yohji Matsusaka, Kenji Fukushima, Akira Seto, Ichiei Kuji, Ichiro Matsunari

**Affiliations:** 1 Division of Nuclear Medicine, Department of Radiology, Saitama Medical University Hospital, 38 Moro-Hongo, Moroyama, 350-0495 Japan; 2 Department of Nuclear Medicine, Saitama Medical University International Medical Center, 1397-1 Yamane, Hidaka, 350-0491 Japan; 3 Department of Central Radiological Technology, Saitama Medical University Hospital, 38 Moro-Hongo, Moroyama, 350-0495 Japan

**Keywords:** Medical imaging, Radionuclide imaging, Molecular medicine, Prostate cancer

## Abstract

The aim of this study was to evaluate the quantitative values of short-time scan (STS) of metastatic lesions compared with a standard scan (SS) when acquired by whole-body bone SPECT/CT with cadmium–zinc–telluride (CZT) detectors. We retrospectively reviewed 13 patients with bone metastases from prostate cancer, who underwent SPECT/CT performed on whole-body CZT gamma cameras. STSs were obtained using 75, 50, 25, 10, and 5% of the list-mode data for SS, respectively. Regions of interest (ROIs) were set on the increased uptake areas diagnosed as metastases. Intraclass correlation coefficients (ICCs) of standardized uptake values (SUVs) for the ROIs were calculated between the SS and each STS, and ICC ≥ 0.8 was set as a perfect correlation. Moreover, the repeatability coefficient (RC) was calculated, and RC ≤ 20% was defined as acceptable. A total of 152 metastatic lesions were included in the analysis. The ICCs between the SS vs. 75%-STS, 50%-STS, 25%-STS, 10%-STS, and 5%-STS were 0.999, 0.997, 0.994, 0.983, and 0.955, respectively. The RCs of the SS vs. 75%-STS, 50%-STS, 25%-STS, 10%-STS, and 5%-STS were 7.9, 12.4, 19.8, 30.8, and 41.3%, respectively. When evaluating the quality of CZT bone SPECT/CT acquired by a standard protocol, 25%-STS may provide adequate quantitative values.

## Introduction

Bone scans using phosphate-based radiopharmaceutical tracers are widely used for the diagnosis of bone disorders. While whole-body imaging has been long-used for bone scans, it is now known that adding single-photon emission computed tomography (SPECT) or SPECT with computed tomography (SPECT/CT) can increase the diagnostic capabilities of whole-body imaging^1,2^. However, the addition of SPECT sequences increases the total scan time. A shorter scan is preferred, especially in patients with painful bone metastases who find it difficult to stay in a single position. Moreover, the feasibility of shorter scan times indicates the possibility of lower dose to the patient when the same scan time is used for SPECT image acquisition.

Gamma cameras with cadmium–zinc–telluride (CZT) detectors have advantages over conventional scintillation cameras, which have increased spatial, time, and energy resolution. Clinical applications of CZT-based SPECT initially began with dedicated cardiac SPECT systems, which were reported to acquire images in a shorter time than conventional systems^3,4^. Subsequently, companies began marketing whole-body SPECT/CT using CZT detectors, and the possibility of a short-time scan (STS) has been reported in cardiac^5^, brain^6,7^, and whole-body bone scan^8^. However, it is as yet unknown whether or not short-time acquisition is acceptable for clinical applications related to SPECT/CT bone imaging using CZT detectors.

Quantitative evaluation is essential for objectively evaluating SPECT uptake values. Techniques for the standardization of these values have been developed using advances in hardware and software^9,10^. Among such techniques, the standardized uptake value (SUV), originally used in positron emission tomography (PET), can be an appropriate representative index even for SPECT. SUVs have recently been utilized in SPECT/CT bone imaging for malignant lesions, such as when differentiating between benign degeneration and metastases^11^, or when evaluating the treatment effects of ^223^Ra-chloride, which is used as radionuclide therapy of bone metastases from prostate cancer^12^. It is also utilized for benign lesions, such as in the evaluation of the clinical stage of antiresorptive agent-related osteonecrosis of the jaw^13^, or in the evaluation of osteoblastic activity in the epiphyseal growth plates of children^14^. Although instability or vulnerability may be associated with SUVs for SPECT in comparison with those for PET, it has been found to be acceptable in most conditions after phantom-base analysis for quality assessment^9,15,16^, or in clinical-based assessments such as test–retest repeatability^17^. Therefore, images acquired in a shorter time might be acceptable for clinical use when the variance of the SUV is within a specified allowance. The present study aimed to evaluate the clinical feasibility of short acquisition times for the bone SPECT/CT using whole-body CZT detectors.

## Methods

### Patients

This retrospective study was conducted in accordance with the guidelines of the Declaration of Helsinki. All experimental protocols in the present study were approved by Institutional Review Board of Saitama Medical University Hospital (No. 20158.01), and the need for written informed consent was waived due to the retrospective nature of the study. From the male patients with bone metastases from prostate cancer who underwent bone scans at our institution between December 2019 and December 2020, we selected 13 patients who met the following criteria: SPECT/CT was acquired using a fixed protocol as shown in the next subsection, and at least one metastatic lesion was confirmed based on the consensus of two experienced nuclear medicine physicians. We estimated 39 lesions to be the minimum required for an adequate analysis of intraclass correlation coefficient (ICC), based on the table from Shoukri et al.^18^, with the parameters of *n* = 2, *α* = 0.05, *β* = 0.20, *ρ*_0_ = 0.6, and *ρ*_1_ = 0.8, where *n* was observations per subject, *α* was the probability of type-I error, *β* was the probability of type-II error, *ρ*_0_ was the ICC when the null hypothesis was true, and *ρ*_1_ is the ICC when the alternative hypothesis was true.

### Bone SPECT/CT protocol: standard scan

Initially, the patients received intravenous injections of ^99m^Tc-methylene diphosphonate (^99m^Tc-MDP). Although 740 MBq was set as the standard target dose, we measured the dose in the syringe before and after administration, and calculated the precise dose injected from these values. Approximately 3 h after injecting the tracer, the patients were asked to void, after which whole-body bone and the associated SPECT/CT images were acquired using a Discovery NM/CT 670 CZT scanner (GE Healthcare, Chicago, IL, USA).

SPECT images for the standard scan (SS) were acquired using the following parameters: a total of 60 projections of 20 s each over 360° in a non-circular orbit, step-and-shoot acquisition by dual-head CZT detectors, high-energy high-resolution collimator, and energy window of 140.5 keV ± 7.5%. It indicated that 10 min was the standard scan time per table position when we did not consider time for gamma-camera rotation. All SPECT images were reconstructed using the ordered subset expectation maximization method, with iteration 4 and subset 10, the matrix size was 128 × 128, and the voxel size was 4.42 × 4.42 × 4.42 mm. CT images were acquired using the following parameters: 120 kV and auto mA (noise index 35) using an ASiR reconstruction system (GE Healthcare), 512 × 512 matrix, 1.375 pitch, and 0.5 s rotation. Although this SPECT/CT system could acquire SPECT data during the rotation of the gamma camera, named as SwiftScan (GE Healthcare), these data were not used in the present study to clarify the analysis.

### Bone SPECT/CT protocol: short-time scan

The SPECT/CT image data were acquired using list-mode, and the images of STSs were reconstructed using 75, 50, 25, 10, and 5% data of SS. The 5%-STS image indicates the shortest acquisition for the scanner, indicating 1 s per step of the gamma cameras.

### Placement of region of interest

We used a workstation with Xeleris v9.0 (GE Healthcare) for region of interest (ROI) placement and SUV calculation. Based on the SS images, each 3-dimensional (3D) ROI was placed to cover the increased uptake interpreted as bone metastasis by the consensus of two board-certified nuclear medicine physicians. The maximum SUV (SUVmax) was calculated for each ROI. The reference ROI, which was healthy bone, was then set on the proximal area of the femur. To choose the reference area, the ROI was set to avoid metastatic lesions.

### Statistics

First, the variance of SUVmax between the SS and each STS was evaluated based on the ICC. Statistical analyses were performed using SPSS 27 (IBM, Armonk, NY, USA). For the calculation of the ICCs, the two-way random model, absolute agreement type, and single measurement data were used in SPSS 27. An ICC ≥ 0.8 was considered as an almost perfect correlation^19^. Second, we drew Bland–Altman plots between the SS and each STS. The repeatability coefficient (RC), reflecting 95% limits of repeatability for the relative difference between the two measurements on the Bland–Altman plot, was calculated to be 1.96 times the standard deviation of relative differences. For the evaluation of RC, ≤ 20% was defined as acceptable for the variation based on the results of previous studies concerning the test–retest repeatability of SUVmax on PET or SPECT images^20–22^. Third, we calculated the rate at which SUVmax changed by ≥ 5% and ≥ 10% for each STS compared with the SS. Finally, we calculated the contrast-to-noise ratio (CNR) using the following formula:

$$ {\text{CNR}} = \frac{{{\text{SUVmax of the lesion}} {-} {\text{SUVmean of the reference}}}}{{\text{Standard deviation of the reference SUV}}}. $$

The CNR of the SS was statistically compared with that of each STS using the Wilcoxon signed-rank test, and a *p*-value of < 0.05 was considered statistically significant.

## Results

### Patients

**Figure 1:**
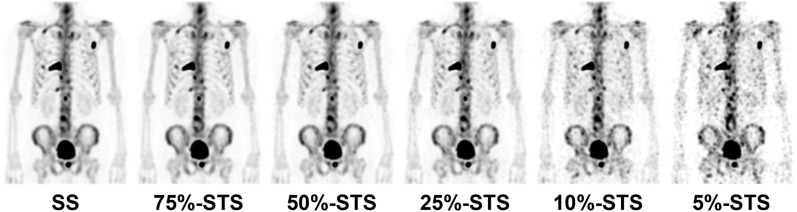
Standard scan (SS) and short-time scan (STS) images of a representative case (#2) with bone metastases in a prostate cancer patient.

A total of 152 metastatic lesions from 13 patients were selected for analysis. Injected dose ranged from 600 to 905 MBq (median 740 MBq), and the time of imaging after injection ranged from 165 to 220 min (median 200 min). The patient characteristics are shown in Table 1, and maximum intensity projection SPECT images of a representative case are shown in Fig. 1.

**Table 1:** Patient characteristics.

Patient no.	Age (years)	Dose (MBq)	SPECT table position number	Time between tracer injection and SPECT scan start (min)	Number of 3D-ROI (s)
1	82	878	3	206	21
2	76	740	2	194	11
3	69	662	2	207	11
4	69	600	1	216	1
5	65	869	3	200	21
6	83	687	2	204	8
7	72	673	2	197	7
8	74	699	2	198	10
9	80	905	1	171	6
10	76	745	1	190	15
11	76	765	2	220	11
12	74	726	2	201	18
13	79	765	2	165	12
Median	76	740	2	200	11

*3D-ROI* three dimensional-region of interest.

### Agreement analysis

**Figure 2:**
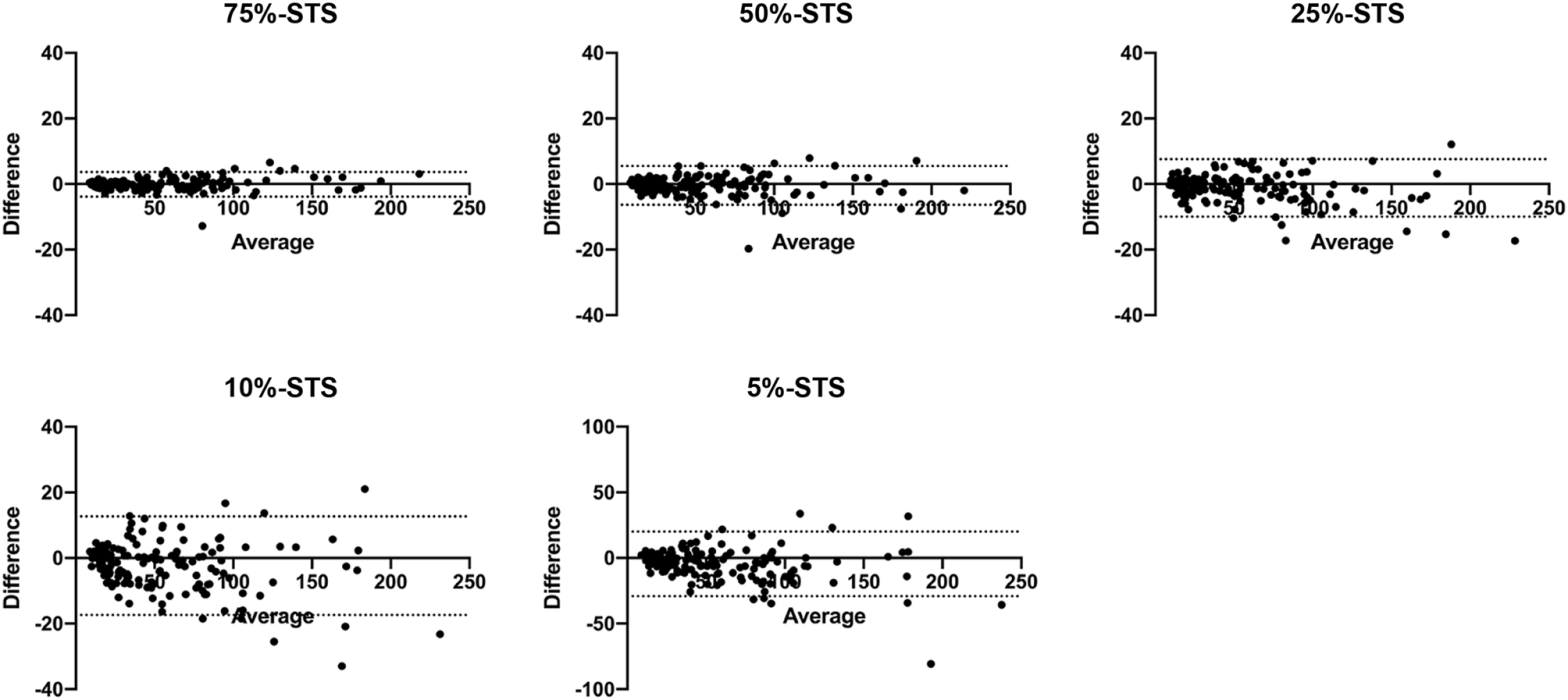
Bland–Altman plots of short-time scan (STS) images. Horizontal dot lines indicate 95%-limits of agreement. Note that the range of difference on the vertical axis for 5%-STS (− 100 to 100%) is different from those in others (− 40 to 40%).

The ICC and RC results are summarized in Table 2. The ICC was 0.955, even in 5%-STS, and all STS images were found to have almost perfect agreement. The Bland–Altman plots are shown in Fig. 2. The RCs between the SS and 75%-STS, 50%-STS, and 25%-STS were < 20% and were therefore found to be acceptable, whereas those between the SS and 10%-STS and 5%-STS were > 20%, and were therefore found to be non-acceptable.

**Table 2:** Intraclass correlation coefficient and repeatability coefficient between standard scan and each short-time scan.

	ICC (95% CI)	RC (%)
SS vs 75%-STS	0.999 (0.999–0.999)	7.9
SS vs 50%-STS	0.997 (0.996–0.998)	12.4
SS vs 25%-STS	0.994 (0.992–0.996)	19.8
SS vs 10%-STS	0.983 (0.975–0.988)	30.8
SS vs 5%-STS	0.955 (0.928–0.970)	41.3

*ICC* intraclass correlation coefficient, *RC* repeatability coefficient, *CI* confidence interval, *SS* standard scan, *STS* short-time scan.

**Figure 3:**
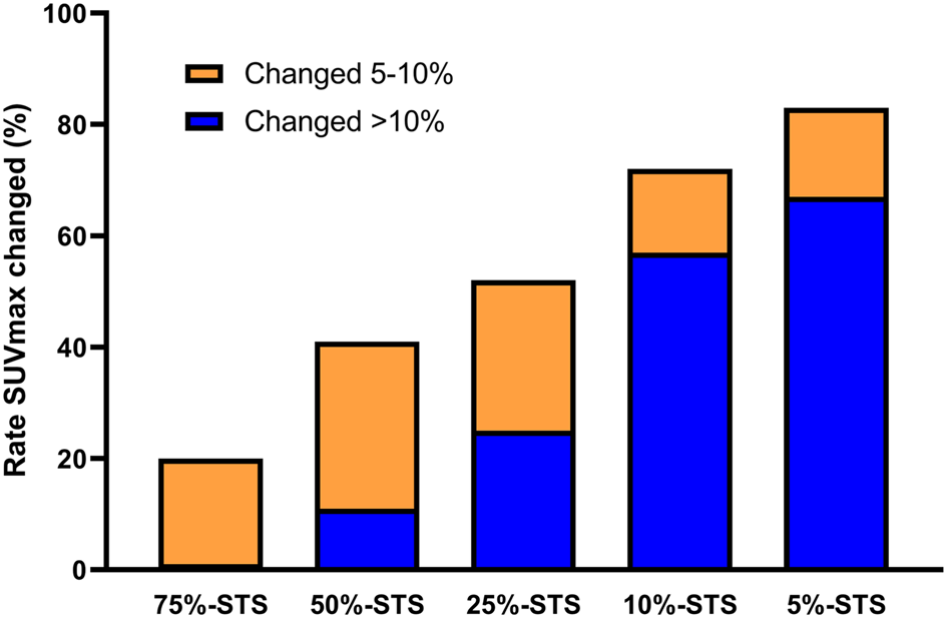
Bar chart for the change in the rate of maximum standardized uptake value (SUVmax) from the standard scan to short-time scan (STS). Rate changes of 5–10% are orange and changes > 10% are blue.

The SUVmax changes are shown in Fig. 3. The rates of SUVmax changes ≥ 10% were 1.3% at 75%-STS, 11.8% at 50%-STS, 25.0% at 25%-STS, 59.7% at 10%-STS, and 67.1% at 5%-STS.

### Contrast-to-noise ratio

**Figure 4:**
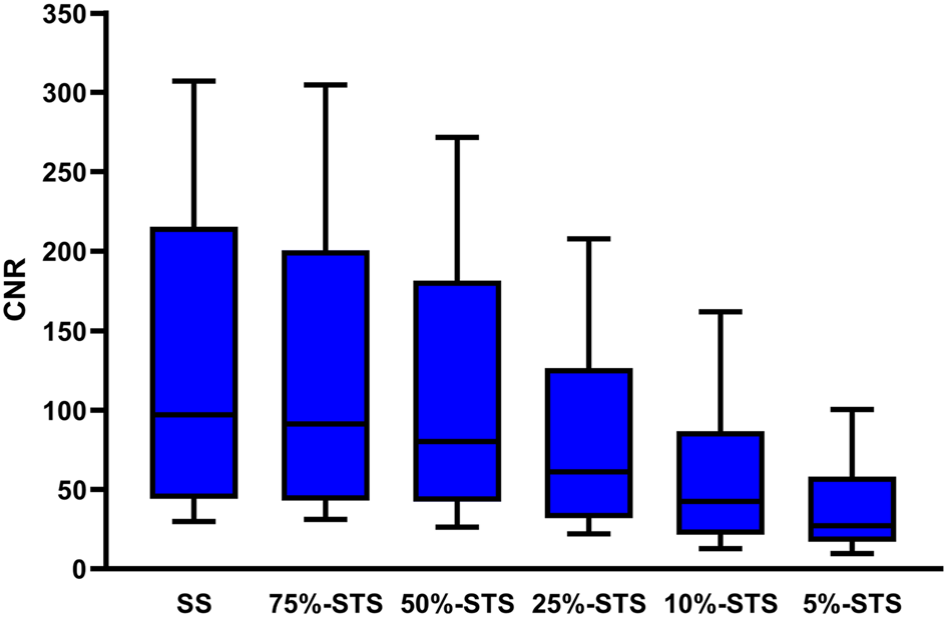
Boxplots of contrast-to-noise ratio (CNR) in standard scans (SSs) and short-time scans (STSs). Lower and upper limits of the whisker indicate the 10th and 90th percentiles of the data. Significant differences were observed between the SS and each STS.

Figure 4 shows the CNRs of the SS and each STS. CNR medians were 97.2, 91.4, 80.1, 61.2, 42.6, and 27.1 for the SS, 75%-STS, 50%-STS, 25%-STS, 10%-STS, and 5%-STS, respectively. A significant difference was observed between the SS and each STS (*p* = 0.04 between the SS and 75%-STS, and *p* < 0.001 between the SS and the other STSs).

## Discussion

To evaluate the clinical applicability of the quantitative values acquired from STS SPECT/CT images, we utilized list-mode data to simulate short-time acquisition of whole-body SPECT/CT bone scans performed in patients with bone metastases from prostate cancer. The resulting ICCs between the SS and each STS showed almost perfect agreement, even between the SS and 5%-STS (1/20th scan time).

ICC is one of the most frequently used indicators to evaluate reproducibility. Although there is no specific cutoff, an ICC of ≥ 0.8^19^ or ≥ 0.75^23^ is generally considered to be an almost perfect correlation, based on the definition of the kappa agreement value. From this point of view, an ICC of 0.955, even between SS and 5%-STS, showed extremely high reproducibility. We also evaluated RC, another absolute indicator for reproducibility, to reinforce the statistical result because some studies recommended using both relative and absolute indicators^24,25^. Although no optimal cutoff was identified on the RC, we set 20% as the cutoff for the present study, based on previous studies that evaluated the reliabilities of SUVs^20–22^. As a result, we found that 25%-STS may be reliable at providing quantitative values. In the 25%-STS images, the SUV changed by ≥ 10% in 25.0% of the lesions. Based on the results of the previous studies regarding repeatability, changes in the SUV may be acceptable at ≤ 10%. As the incidence of the SUV change dramatically increases in the 10%-STS, the 25%-STS may be the shortest with which to maintain the reliability of quantitative values.

Contrarily, background noise gradually increased as the scan time decreased. Statistically significant differences were observed in the CNR between the SS and STS, even in 75%-STS, which may lead to false-positive results in the interpretation of SPECT/CT bone scans when using a shorter acquisition time. There may be a discrepancy in the results of ICC or RC analysis. Generally, uptake on bone scans in bone metastases is considered quite high^11^ compared with that in other types of metastatic or bone disorders^26^. Such a high quantitative value, therefore, may have been unaffected by the decrease in scan time and the subsequent noise on the images. These results are quite similar to those from the STS of a planar bone scan^8^. As a result, we feel that 25%-STS provides appropriate quantitative values for whole-body SPECT/CT scans for bone metastases from prostate cancer. Reducing scan time enables patients with pain to shorten the restrain. Instead of scan time, our results can apply for reducing tracer dose. For patients who need multiple follow-up scans, the benefits of low-dose scans must be significant. However, short acquisition time results in the reduction of image quality. The shorter the image acquisition time was, the more SUVmax changed, and the lower CNR was. On the other hand, more acquisition time, like > 75%, may be required for the initial diagnosis, even in the case of bone metastases from prostate cancer, or other types of bone metastasis or bone disorders that have lower uptakes. Therefore, we should use proper acquisition time or tracer dose for what is essential for the patient and how much the image quality is required. In addition, we should note that the median tracer dose we used in the present study was 740 MBq, which is relatively high when we consider 555 MBq as the standard dose. In that case, more acquisition time may be required for the appropriate quantification. Further studies are needed to evaluate the reliability of STS in different patient groups, including other malignant tumors or bone disorders.

Our proposed methods for short-time acquisition may be helpful to develop dynamic quantitative SPECT/CT imaging, so far a domain of PET. Tracers with more complex kinetics, such as ^99m^Tc-sestamibi or even new radiopharmaceuticals, could be evaluated. In addition to ^99m^Tc labeled tracer, the development of new collimators of wide-energy high-resolution or medium-energy high-resolution can acquire images of other radionuclides, including ^123^I or ^177^Lu, that can apply for the dosimetry of radionuclide therapy^27^.

One limitation of the present study was that we did not evaluate the diagnostic capabilities of these scans, including sensitivity and specificity. Although we chose metastatic lesions based on the consensus of experienced nuclear medicine doctors, the results were not confirmed by pathological methods. Therefore, we can only discuss the quantitative values as the results of the present study.

In conclusion, regarding bone SPECT/CT images acquired using a standard protocol of 740 MBq tracer dose and 10 min/bed position, we found that 25%-STS might be acceptable for the clinical evaluation of the quantitative values of bone whole-body SPECT/CT images acquired using CZT detectors, specifically for bone metastases from prostate cancer.
